# Comprehensive Morphological and Immunohistochemical Evaluation of Gastrointestinal Stromal Tumors: Insights From a Tertiary Care Center

**DOI:** 10.7759/cureus.108073

**Published:** 2026-04-30

**Authors:** Vaishali Pol, Mahendra Patil, Vivek Dugad, Jaydeep N Pol, Anand Bhosale, Ashna Agarwal, Pranjali Deshpande

**Affiliations:** 1 Pathology, Deep Pathology Laboratory, Miraj, IND; 2 Pathology, Krishna Vishwa Vidyapeeth (Deemed to be University), Karad, IND; 3 Pathology, DY Patil University School of Medicine and Pushpalata DY Patil Hospital, Pune, IND; 4 Pathology, Mahatma Gandhi Cancer Hospital, Miraj, IND; 5 Physiology, DY Patil University School of Medicine and Pushpalata DY Patil Hospital, Pune, IND

**Keywords:** cd117, cd34, dog1, gastrointestinal stromal tumors, histopathology, immunohistochemistry, soft tissue

## Abstract

Introduction

Gastrointestinal stromal tumors (GISTs) represent the most common mesenchymal neoplasms of the gastrointestinal tract, typically originating from the interstitial cells of Cajal. Accurate histopathological assessment and immunohistochemical profiling are pivotal for diagnosis and risk stratification. This study aimed to evaluate the clinicopathological characteristics and immunohistochemical expression patterns of GISTs diagnosed at a tertiary care center.

Methods

A retrospective analysis was conducted on 30 histologically confirmed cases of GIST over a seven-year period. Formalin-fixed, paraffin-embedded tissue samples were reviewed using hematoxylin and eosin staining and subjected to immunohistochemistry. Parameters assessed included patient demographics, tumor location, histological subtype, mitotic index, and immunoreactivity for CD117, DOG1, and CD34.

Results

The cohort comprised 19 men and 11 women, with a mean age of 62.1 years. The small intestine had the most number of cases, i.e., eight cases (26%), followed by the stomach, which had seven cases (24%). Spindle cell morphology was predominant (86%), followed by epithelioid and mixed patterns (7% each). Most tumors were low-grade (60%), with a mitotic rate of <5 per 50 high-power fields. Immunohistochemically, DOG1 showed the highest positivity (97%), followed by CD117 (84%) and CD34 (70%). Smooth muscle actin (SMA), desmin, and S-100 showed variable positivity.

Conclusion

This study highlights a male predominance and a predilection for gastric and small intestinal involvement in GISTs. Spindle cell morphology and low mitotic activity were common histological features. Immunohistochemistry remains indispensable for diagnosis, with DOG1 demonstrating superior sensitivity. These findings reinforce the critical role of integrated histopathological and immunohistochemical evaluation in the accurate diagnosis and management of GISTs.

## Introduction

In 1940, Golden described stromal tumors arising from the gastrointestinal tract smooth muscle as leiomyoma, leiomyosarcoma, leiomyoblastoma, and bizarre leiomyoma [1]. The term gastrointestinal stromal tumors (GISTs) was first used in 1983 by Mazur and Clark, and in 1998, a Japanese researcher discovered the presence of a KIT protein and a possibility of KIT mutations, which distinguishes GIST from smooth muscle tumors [2]. Eighty-five percent of the GISTs have an active mutation in the KIT protooncogene, while only 3%-5% have a mutation in PDGFRA [2]. With the identification of immunopositivity to CD34 and CD117 (c-KIT protein), GISTs are the predominant primary non-epithelial tumors of the gastrointestinal tract. These tumors are common in older individuals, having a median age of 55-65 years. GIST usually originates from interstitial cells of Cajal, a site in the muscularis propria and in the gastrointestinal myenteric plexus, known as the pacemaker cells of bowel peristalsis [3]. Accounting for approximately 0.2% of all gastrointestinal tumors, they most commonly develop in the stomach (40%-70%) and small intestine (20%-50%) [4]. These tumors typically emerge between the fourth and sixth decades of life. A hallmark of GISTs is the overexpression of the tyrosine kinase receptor, a protein product of the C-KIT gene (KIT) [4,5]. Other tumors that also show overexpression of tyrosine kinase receptors are non-small-cell lung carcinoma, breast carcinoma, melanoma, and chronic myeloid leukemia. Tumor size and mitotic activity serve as critical prognostic factors in assessing the risk of aggressive tumor behavior [4,5]. The mainstay of treatment for GIST is surgical resection of the tumor, but the results of surgery alone have been inadequate, with up to 50% of the patients developing tumor recurrence within the first five years. Post-operative chemotherapy is also ineffective. The use of small-molecule kinase inhibitors that target the underlying pathogenic mutant is used for the treatment of GIST [2,3]. There are many significant research gaps for a better understanding of the disease, such as secondary mutations in the KIT or PDGFRA protein, which necessitate alternate therapy. Despite the risk stratification systems, predicting recurrence and metastasis remains imprecise. This study aimed to analyze the histomorphological and immunohistochemical (IHC) correlation in a soft tissue mass for diagnosing GIST.

## Materials and methods

This retrospective observational study was conducted in the Department of Pathology at a tertiary care center (Mahatma Gandhi Cancer Hospital, Miraj, India) over a seven-year period from January 2018 to October 2025. A total of 30 cases of GISTs, confirmed by histopathological and IHC evaluation, were included.

The study received approval from the Institutional Ethics Committee and adhered to the principles of the Declaration of Helsinki. Written informed consent was obtained from all the patients.

Inclusion and exclusion criteria

The inclusion criteria include cases clinically, radiologically, and histologically suspected and IHC confirmed as GIST. The exclusion criteria include cases suspected clinically or histologically but not confirmed as GISTs.

Methods

Complete clinical history, physical examination findings, and radiological investigation findings were recorded. Tissue specimens from excisional biopsies were fixed in 10% neutral-buffered formalin. Gross examination included assessment of tumor size, consistency, cut surface, and areas of hemorrhage and necrosis. Representative sections (3-4 mm) were processed, embedded in paraffin, and stained with hematoxylin and eosin for microscopic evaluation of the GISTs.

IHC analysis was performed on formalin-fixed paraffin-embedded sections using a BioGenex automated antigen-retrieval and auto-stainer system. Primary antibodies include CD117 (clone YR145, Rabbit Monoclonal, Cell Marque), DOG1 (clone 1.1, mouse monoclonal, BioGenex), and CD34 (clone QBEnd/10, mouse monoclonal, BioGenex). All antibodies were undiluted. Appropriate positive and negative controls were also used. Other stains that were also used for differentiating GIST from leiomyoma, schwannoma, and sarcoma were smooth muscle actin (SMA), desmin, S-100, and calretinin. Staining was interpreted as follows: CD117 membranous, cytoplasmic, or perinuclear-dot positivity, DOG1 as membranous and cytoplasmic positivity, and CD34 as membranous positivity.

The pathologic diagnosis of the GIST depends on the morphological and IHC findings. KIT and DOG1 are the two most important markers; DOG1 is the most specific marker, and CKIT serves as the predictive marker for target therapy for tyrosine kinase inhibitors like imatinib. When KIT is negative, other markers such as DOG1 followed by CD34 are also diagnostic. The criteria used for histological grading and risk stratification were the Miettinen and Lasota criteria, which incorporated the anatomic site of location of the tumor, tumor size, and mitotic count [6,7].

Statistical analysis

All data were entered into Microsoft Excel 2021 (Microsoft Corp., Redmond, WA, USA) and analyzed using descriptive statistics including mean, median, frequency, and percentage. Results were presented in tables and graphs to summarize clinicopathological characteristics and IHC findings.

## Results

A total of 30 cases were identified, indicating a male predominance with a male-to-female ratio of 1.73:1. The patient age ranged from 43 to 87 years, with a mean age of 62.1 years and a median age of 62.5 years. The highest proportion of cases was in the age group 60-70 years, having 10 cases (33.33%). Table 1 shows the age- and gender-wise distribution of the cases.

**Table 1:** Age- and gender-wise distribution of GIST cases. GIST: gastrointestinal stromal tumor

Age range	Males	Females	No. of cases	Percentage
40-50	2	2	4	13.34%
50-60	6	3	9	30.00%
60-70	7	3	10	33.33%
70-80	2	2	4	13.33%
>80	2	1	3	10.00%
Total	19	11	30	100.00%

The most common sites of gastrointestinal tumors were the stomach, with seven cases (24%), and the small intestine, with eight cases (26%), followed by the rectum and pelvis, with three cases (10%) each. The peritoneum, omentum, and epigastric regions combined have four cases (14%), whereas the right hypochondriac had two cases (7%). The abdomen, bowel wall, and left lumbar have one case (3%) each. Table 2 shows the distribution of GIST cases at different sites.

**Table 2:** Site-specific frequency of GIST. GIST: gastrointestinal stromal tumor

Site of presentation	No. of cases	Percentage
Abdomen	1	3.00%
Peritoneum, omentum, epigastric region	4	14.00%
Rectum, right lateral wall, anterior wall	3	10.00%
Stomach, body of stomach, lesser curvature, serosal surface	7	24.00%
Small intestine, ileum, jejunum, duodenum, jejunum junction, periampullary region	8	26.00%
Bowel wall	1	3.00%
Left lumbar	1	3.00%
Pelvis	3	10.00%
Right hypochondriac	2	7.00%
Total	30	100.00%

**Figure 1:**
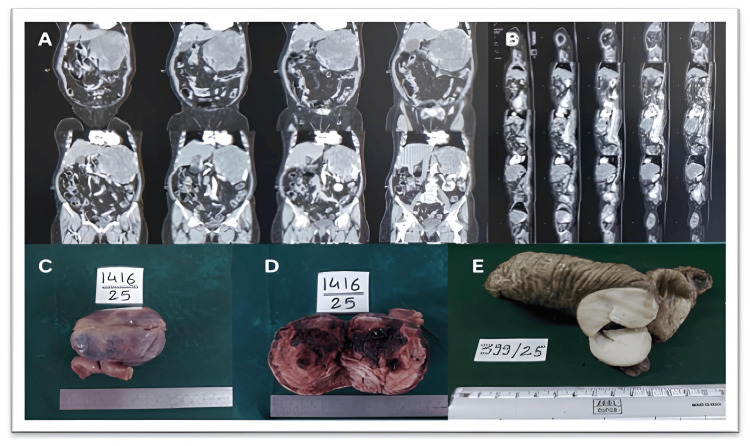
(A, B) Coronal and sagittal views of the CT scan of case 28 showing a large exophytic soft tissue mass epicentered in the stomach. Central areas of necrosis are seen with heterogeneous post-contrast enhancement. (C, D) Gross images of case 25 showing the ileum with a grayish-white mass. The cut section shows areas of hemorrhage and necrosis. (E) Gross image of case 12 showing the intestine with a solid whitish mass having smooth margins on the cut section.

**Figure 2:**
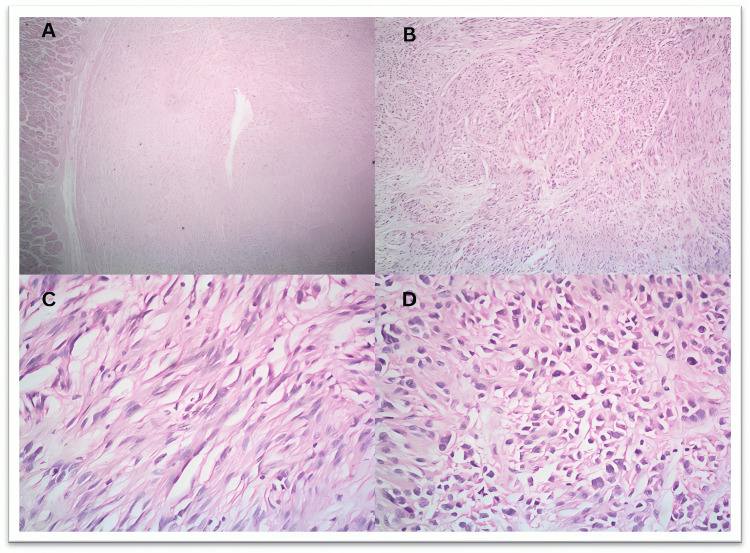
Microscopic image of case no. 21 showing (A) a submucosal well-circumscribed tumor in the stomach (H&E 4x). (B, C) Spindle and epithelioid cell morphology arranged in fascicles, which are densely packed: (B) H&E 10x; (C) H&E 40x. (D) Predominantly epithelioid cells with marked nuclear atypia and few mitotic figures (H&E 40x). H&E: hematoxylin and eosin

**Figure 3:**
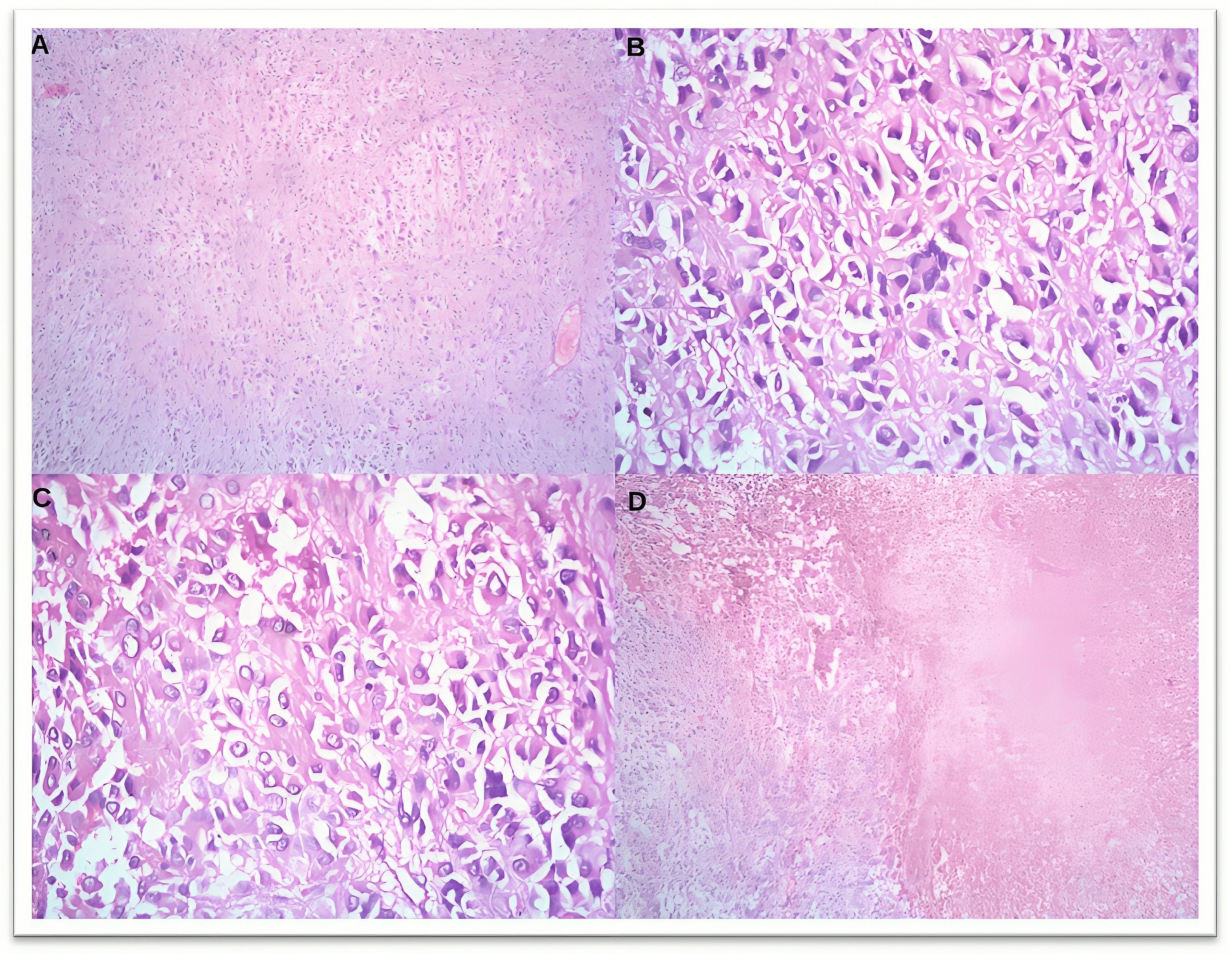
Microscopic images of case 24 showing a high-grade GIST with epithelioid morphology. The tumor shows (A) predominantly epithelioid cells (10x), (B) marked nuclear atypia with multinucleation (40x), (C) increased mitosis (40x), and (D) areas of hemorrhage and necrosis (10x). GIST: gastrointestinal stromal tumor

**Figure 4:**
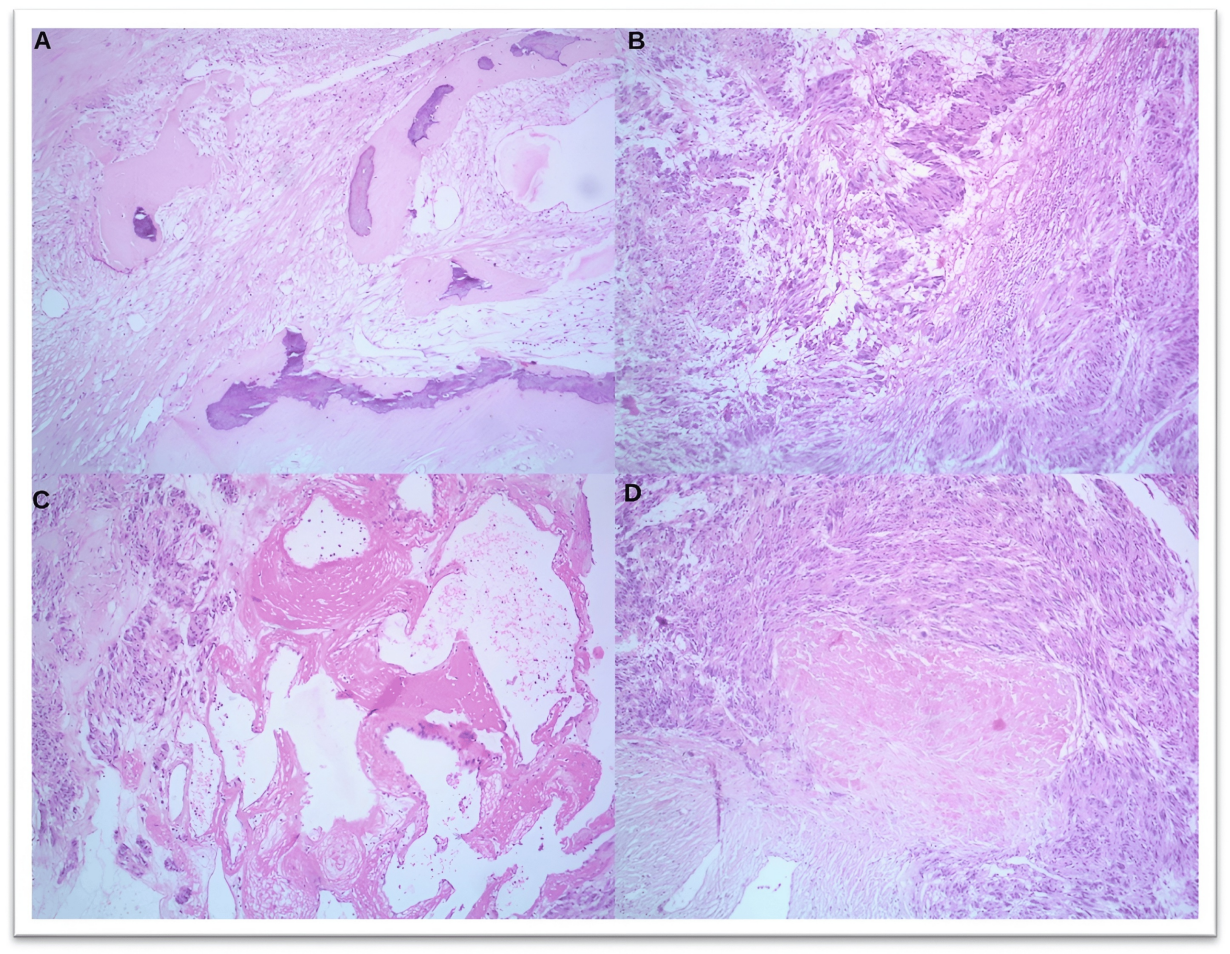
H&E images of GIST cases showing various morphological variations like (A) osseous metaplasia (10x), (B) schwannoma-like areas with Verocay body-like structures (10x), (C) areas of hemorrhage (10x), and (D) infarction (10x). GIST: gastrointestinal stromal tumor; H&E: hematoxylin and eosin

**Figure 5:**
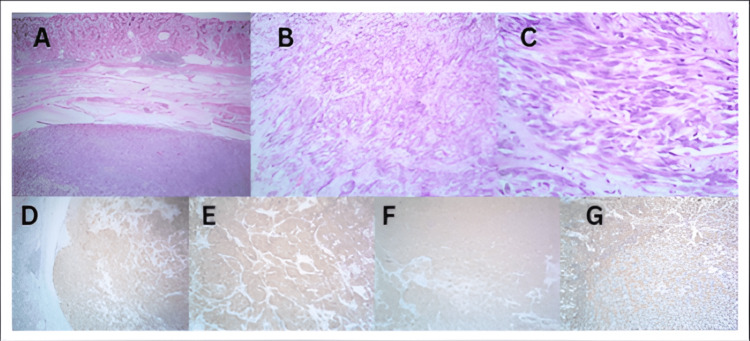
Microscopic images of case 13 showing (A) a submucosal tumor in the stomach with intact mucosa (H&E 4x), (B) spindle cell morphology (H&E 10x), and (C) minimal nuclear atypia (H&E 40x). (D-G) Immunohistochemistry images of case 13 showing strong and diffuse expression of (D, E) CKIT (CD117) (D, 4x; E, 10x), (F) CD34 (10x), and (G) DOG1 (10x). H&E: hematoxylin and eosin

**Figure 6:**
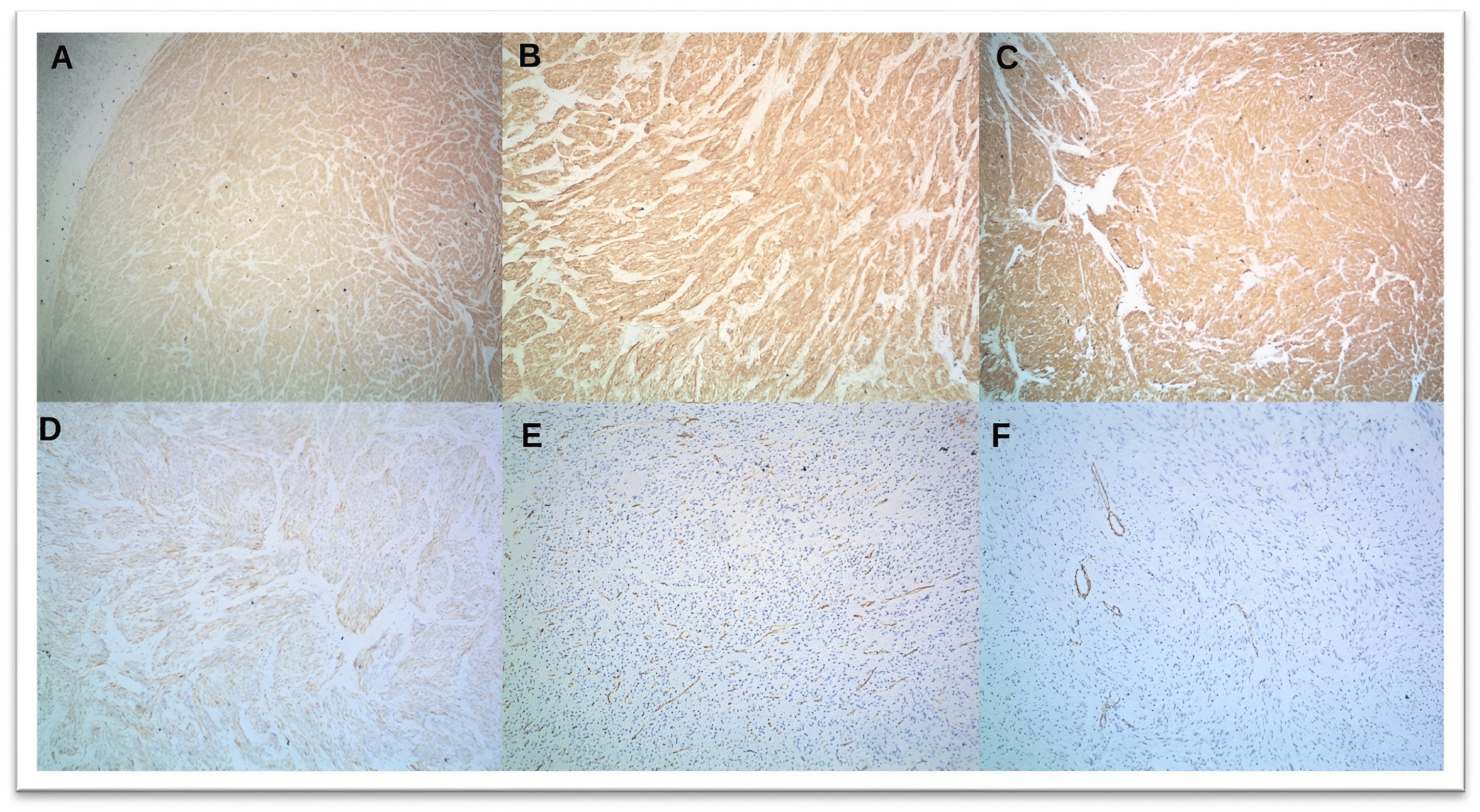
Immunohistochemistry images of case 21 showing strong and diffuse expression of (A, B) CKIT (CD117) (A, 4x; B, 10x). (C) DOG1 (10x) and (D) desmin (10x). The tumor cells are negative for (E) CD34 (10x) and (F) SMA (10x). SMA: smooth muscle actin

**Figure 7:**
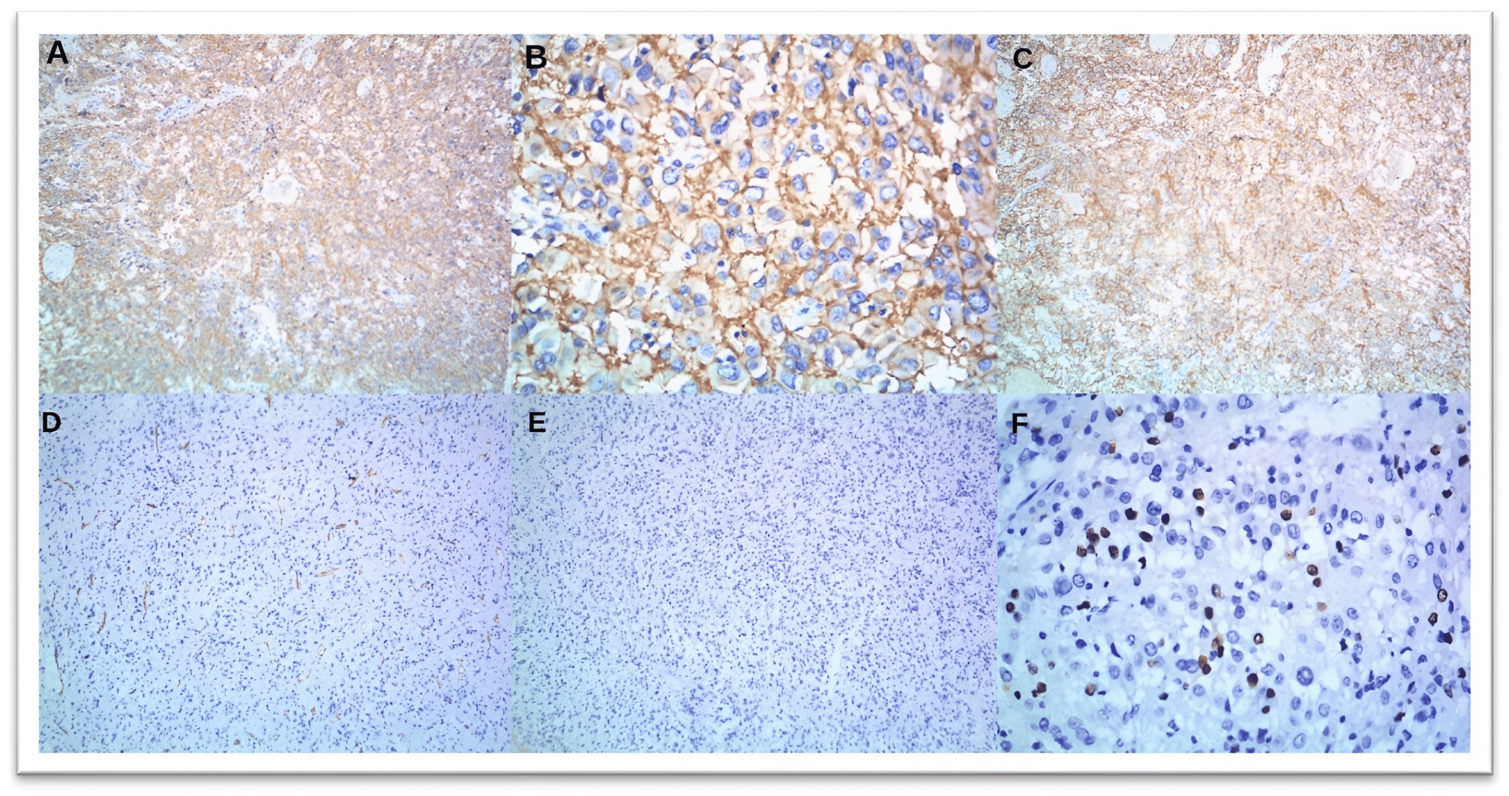
Immunohistochemistry images of case 24 showing strong and diffuse expression of (A, B) CKIT (CD117) (A, 10x; B, 40x) and (C) DOG1 (10x). The tumor cells are negative for (D) CD34 (10x) and (E) desmin (10x), and (F) Mib1 proliferation index is around 10% (40x).

On macroscopic examination, the tumor size in the present study varied from the smallest being 2.5 cm to the largest being 19 cm. Macroscopic pictures are depicted in Figures 1C-1E of case numbers 25 and 12, along with the coronal and sagittal views of the CT scan of case 28. The microscopic images of various cases, along with relevant IHC from our study, are shown in Figures 2-7. Microscopy of the GIST showed different morphologies, the most common being the spindle cell seen in 26 cases (86.66%), arranged in fascicles. Two cases, i.e., 6.67%, had an epithelioid cell type, which showed epithelial-like cells having eosinophilic cytoplasm and had necrosis and hemorrhage in the background. Two cases had a mixed type of morphology (6.67%), with features of both spindle cell and epithelioid cell arranged in fascicles. Table 3 shows different morphologies observed in the study.

**Table 3:** Distribution of cellular morphology in GIST cases. GIST: gastrointestinal stromal tumor

Type of morphology	No. of cases	Percentage
Spindle cell	26	86.66%
Epithelioid cell	2	6.67%
Mixed	2	6.67%
Total	30	100.00%

Tumors were also histologically graded into low-grade, with 18 cases (60.0%), as shown in Figure 5, and high-grade, with 12 cases (40.0%), as shown in Figures 2, 3. Table 4 shows the histological grading of the cases. GIST also showed various morphological variations. One case showed osseous metaplasia, two cases showed schwannoma-like verrucae bodies, whereas two other areas showed infarction, and one case had hemangioma-like areas, as shown in Figure 4.

**Table 4:** Histological grading of GIST cases. GIST: gastrointestinal stromal tumor

Histological grade	No of cases	Percentage
Low grade	18	60.00%
High grade	12	40.00%
Total	30	100.00%

The mitotic rate was also studied, with 19 cases (64%) having <5/50 hpf; eight cases (25%) had 5-20/50 hpf, whereas three cases (11%) had >25-50/50 hpf. There were many differential diagnoses that were being considered for the mass, which included lymphoma, spindle cell neoplasm, sarcoma, leiomyoma, carcinoma stomach, metastatic carcinoma, and GIST. Various IHC markers were used for making the final diagnosis of GIST such as C-KIT (CD117), DOG-, CD34, SMA, desmin, Vimentin, S-100, cytokeratin, proliferation index such as Mib, H caldesmon, calretinin, inhibin, estrogen receptor, beta catenin, arginase, p63, PAX 8, Ca 19.9, CD 31, myogenin, and Myo D1. In the present study, CKIT showed positivity in 25 cases (84%), DOG1 was positive in 29 cases (97%), and CD34 was positive in 21 cases (70%), whereas SMA, desmin, and S-100 showed variable expression, as shown in Figures 2, 6, 7. Out of 30 cases, 18 cases (60%) were positive for all three markers, i.e., DOG1, CKIT, and CD34, whereas 12 cases (40%) were positive for two markers, i.e., CKIT and DOG1, CD34 and DOG1, and CKIT and CD34. Other markers were used to differentiate GIST from other differentials. The diagnosis of GIST was made with the help of histopathological and IHC examination.

## Discussion

GISTs arise within the walls of the gastrointestinal tract, primarily in the stomach, though they also frequently occur in the small intestine and colon. The average annual incidence is 0.32 per 100,000, with a 15-year prevalence of 1.62 per 100,000 [8].

Historically, localized GISTs were managed using surgery and chemotherapy, but chemotherapy has since been proven ineffective, making surgical resection the standard treatment for resectable tumors [8]. These tumors predominantly affect individuals aged 40 to 60 years, with rare cases observed in pediatric patients. The present study showed that the maximum number of cases was present in men (n = 19), and women only had 11 cases, showing male preponderance, which is in concordance with the study conducted by Yacob et al. [9] and Li et al. [10] and differs from the study conducted by Ahmed et al. [11]. The mean age in the present study is 62.1 years, which is different from the study conducted by Ahmed et al. [11] and Tepeoğlu et al. There is no sex predilection for GIST. Their distribution is as follows: stomach (60%), jejunum and ileum (30%), duodenum (5%), colorectum (4%), and esophagus or appendix (1%). In the present study, the most common site of tumor presentation was the stomach (n = 7; 24%) and small intestine (n = 8; 26%), which was different from the study conducted by Søreide et al., as it showed gastric predominance. Clinically, patients may present with bleeding, perforation, or obstruction, though some remain asymptomatic, with tumors detected incidentally or during endoscopy. In the present study, the patients presented with abdominal pain and the presence of a mass in the abdomen and extra-abdominal areas, and a few patients also presented with bleeding and obstruction. The development of GISTs is driven by mutations in the KIT and PDGFRA oncogenes, leading to the activation of the KIT receptor tyrosine kinase. These mutations occur in either the cytoplasmic kinase domains or the juxtamembrane regions, affecting intracellular or extracellular signaling. Most KIT mutations are found in the juxtamembrane region, primarily on exon 11 (≈70%), with a smaller proportion on exon 9 (≈10%). These alterations result in constitutive receptor activation, disrupting normal cellular signaling and promoting unchecked cell proliferation. While KIT and PDGFRA mutations are mutually exclusive, both contribute to GIST oncogenesis by influencing similar cellular signaling pathways, though they act at different receptor sites. PDGFRA mutations occur in a minority of cases (<10%), typically affecting exon 14 or exon 18. These tumors are generally restricted to the stomach, exhibit epithelioid morphology, and tend to be less aggressive [2,5,12-16].

DOG1 (discovered on GIST-1) has been reported to exhibit high sensitivity and specificity in diagnosing GISTs [17]. It is identified as a gene located in the CCDN1-EMS1 locus on human chromosome 11q13 and serves as a reliable IHC marker for detecting GISTs [18].

While most GISTs occur sporadically, they have also been found in association with neurofibromatosis type 1 (NF-1), Carney triad, and Carney-Stratakis syndrome [17]. Carney triad syndrome is defined by the presence of gastric GIST, extra-adrenal paraganglioma, and pulmonary chondroma, whereas Carney-Stratakis syndrome is characterized by gastric GIST and paragangliomas [19].

GISTs linked to Carney triad and Carney-Stratakis syndrome exhibit distinct clinical features compared to sporadic cases, including a female predominance, frequent metastasis, younger age of onset, multifocal presentation, slow growth, resistance to imatinib therapy, and poor prognosis. These tumors typically arise in the gastric antrum and do not harbor KIT or PDGFRA mutations [20-23].

The wild-type variant of GIST is more commonly seen in children (≈85%-90%), whereas only ≈10%-15% of adults exhibit this mutation profile [24]. Wild-type GISTs lack KIT and PDGFRA mutations, and although their exact pathogenesis remains unclear, studies suggest that germline mutations in succinate dehydrogenase (SDH)-particularly in the SDHB and SDHC subunit genes-result in either complete loss or reduced SDH protein expression [25,26].

The study conducted by Lakshmaiah et al. [27] studied 44 cases at a single oncology institute in South India between 2005 and 2011. The majority of the patients were middle-aged men, which is similar to the present study, with the stomach being the most common site. Most of the cases had localized disease, while the rest presented with metastasis, primarily to the liver. All patients received imatinib as adjuvant or palliative therapy. The study highlights that Indian patients often present with bulky tumors and advanced disease.

The retrospective study by Malik et al. [28] analyzed 63 cases of GIST between the period of 1999 and 2012. The stomach was the most common site, and 38 patients had non-metastatic disease. The authors identified that key prognostic factors such as high mitotic rate, non-gastric primary site, male gender, epithelioid histology, and presence of metastasis were significantly associated with poorer overall survival. Multivariate analysis confirmed that high mitotic activity and non-gastric origin independently predicted worse outcomes. The study underscores the rarity of GISTs in India and the need for long-term follow-up and standardized staging to improve prognostication and treatment strategies.

Macroscopically, GISTs originate from the muscularis propria layer of the gastrointestinal tract. These tumors typically exhibit a fleshy pink or tan-white appearance, often accompanied by areas of hemorrhage, necrosis, or cystic degenerative changes [24]. Microscopically, GISTs are relatively uniform tumors that can be classified into three distinct morphological subtypes. The spindle cell type is the most prevalent, accounting for approximately 70% of cases, followed by the epithelioid cell type (20%) and the mixed cell type (10%). The spindle cell variant is characterized by cells with pale eosinophilic fibrillary cytoplasm, ovoid nuclei, and syncytial cell borders, arranged in fascicles or whorls. Vacuolization is frequently observed, along with extracellular deposits of dense collagen, referred to as skeinoid fibers, which are commonly associated with NF-1-related GISTs. The epithelioid cell variant consists of round epithelial-like cells with pale to clear eosinophilic cytoplasm, arranged in nests, sheets, and occasionally cords. This subtype is most frequently encountered in pediatric GISTs. GISTs exhibit variable cellularity and may present with sclerotic, collagenous, or myxoid stromal changes. Although pleomorphism is rare, it may occasionally be observed in certain cases [19].

In our study, 12 cases had a tumor size of more than 10 cm, whereas 16 cases had a tumor size below 10 cm, which is similar to the study conducted by Yacob et al. [9] and Alqusous et al. [29]. The predominant morphology observed in our study was the spindle cell type, which was present in 26 cases, followed by the epithelioid cell type and mixed type, which was in concordance with the study conducted by Li et al. [10] and Vij et al. [30]. In the present study, most of the cases were low-grade, which was similar to the study conducted by Yacob et al. [9] and different from the study conducted by Alqusous et al. [29], which had a larger number of cases in the high grade. In our study, we observed morphological variations such as osseous metaplasia, schwannoma-like verrucae bodies, infarction-like areas, and hemangioma-like areas in a few cases.

The systematic review conducted by Søreide et al. [12] examines global epidemiological data on GISTs from 29 population-based cohort studies across 19 countries. It reveals that GISTs most commonly occur in the stomach and small intestine, which was similar to our study, with a median diagnosis age in the mid-60s and equal gender distribution. Incidental detection is frequent, and reported incidence rates vary widely-from 4.3 to over 20 cases per million annually-highlighting disparities in healthcare access, diagnostic practices, and registry quality. The authors emphasize the need for standardized coding and global data harmonization to improve the understanding and management of GISTs worldwide.

IHC by KIT (CD117) serves as a specific and sensitive marker for GISTs in differential diagnostic settings. Over 90% of GISTs exhibit immunoreactivity for KIT [24]. In most cases, strong and diffuse cytoplasmic staining of KIT is observed, while a small subset of tumors displays a dot-like or membranous staining pattern [13,31-33]. DOG1 is particularly immunoreactive in pediatric GISTs and those associated with NF-1 [34]. CD34, an early marker for GIST, is commonly utilized in the IHC evaluation of spindle cell tumors, although it demonstrates lower sensitivity compared to KIT and DOG1 [35]. Antibodies targeting PDGFRA, a tyrosine kinase receptor closely related to KIT, are useful in cases of KIT-negative GISTs harboring PDGFRA mutations. Strong PDGFRA immunoreactivity is frequently observed in epithelioid GISTs [36]. Additional IHC markers include S-100, caldesmon, SMA, and desmin, though their immunoreactivity varies among different GIST subtypes [19]. In the present study, 84% cases were positive for CD117 (C KIT), DOG1 had 97% positivity, and CD34 had 70% positive cases, which was similar to the study conducted by Alqusous et al. [29]. We also observed the variable positivity of other markers such as S-100, caldesmon, SMA, and desmin among different subtypes of GIST.

The differential diagnosis of GISTs is primarily determined by tumor morphology. For spindle cell type GISTs, the differential diagnoses include leiomyoma, leiomyosarcoma, schwannoma, intra-abdominal desmoid fibromatoses, and solitary fibrous tumor [37]. Leiomyomas and leiomyosarcomas are smooth muscle tumors characterized by spindle-shaped cells with cigar-shaped nuclei, bright eosinophilic cytoplasm, and well-defined cell borders, which contrasts with the ovoid nuclei and syncytial appearance observed in GISTs [31,37]. IHC constitutes the most definitive diagnostic modality for distinguishing GISTs from true smooth muscle neoplasms. GISTs characteristically demonstrate strong and diffuse cytoplasmic positivity for CD117 (KIT) in approximately 95% of cases, reflecting underlying activating mutations in the KIT gene, and also exhibit high sensitivity for DOG1, which is particularly valuable in CD117-negative cases. CD34 expression is observed in a significant proportion of GISTs, although it lacks specificity. In contrast, smooth muscle tumors such as leiomyomas and leiomyosarcomas are typically negative for CD117 and DOG1 but show diffuse cytoplasmic positivity for smooth muscle markers, including SMA and desmin, indicating true myogenic differentiation. Desmin, in particular, is a robust marker favoring smooth muscle origin and is only rarely expressed in GISTs. S-100 protein may show focal positivity in a minority of GISTs but is not a defining feature.

However, certain limitations must be acknowledged. The relatively small sample size (n = 30) limits the generalizability of the findings. Being a single-center retrospective study, there is potential for selection bias and incomplete data retrieval. Molecular analysis, including KIT and PDGFRA mutation testing, was not performed due to financial constraints, thereby limiting detailed prognostic and therapeutic correlations. Furthermore, the absence of long-term follow-up data restricts the assessment of recurrence, metastasis, and survival outcomes. Lastly, the use of primarily descriptive statistical methods limits the ability to establish significant associations between clinicopathological variables. For epithelioid-type GISTs, potential differential diagnoses include neuroendocrine carcinoma and other mesenchymal neoplasms, such as clear cell sarcomas, which can be differentiated by IHC, such as S-100, CK, and Vimentin [38,39].

The risk assessment for GIST recurrence is currently based on tumor morphology [40]. Studies conducted in 2007 and 2010 by the National Comprehensive Cancer Network (NCCN) and the European Organization for Research and Treatment of Cancer (EORTC) recommend that mitotic activity, tumor size, and anatomic location be the primary factors for evaluating recurrence risk [24,41-45].

## Conclusions

GISTs are the most common malignant mesenchymal tumors of the gastrointestinal tract. Accurate differentiation of mesenchymal lesions is crucial for diagnosing GISTs and excluding their morphological mimics because the treatment strategy is quite different. Ancillary techniques such as IHC and molecular analysis serve as valuable tools for confirming the diagnosis.

Histopathological and IHC evaluation play a critical role in assessing patient prognosis, highlighting the essential role of pathologists in the effective management of GISTs. However, molecular analysis was not performed in these cases due to financial constraints.
